# Does Chronic Neck Pain Matter? Auditory Function in Individuals With Forward Head Posture: An Exploratory Cross‐Sectional Comparative Study

**DOI:** 10.1002/brb3.71611

**Published:** 2026-07-16

**Authors:** Kaan Tuğberk Özdemir, Emre Söylemez

**Affiliations:** ^1^ Department of Neurosurgery Medicana International Izmir Hospital Izmir Türkiye; ^2^ Department of Audiometry, Vocational School of Health Services Karabuk University Karabük Türkiye

**Keywords:** auditory function, forward head posture, hearing, neck pain, tinnitus

## Abstract

**Background:**

Evidence suggests that neck pain and cervical pathologies may contribute to auditory disorders; however, auditory functions in individuals with forward head posture (FHP) have been insufficiently studied. This study aimed to explore hearing thresholds, self‐reported auditory difficulties, central auditory processing measures, and the presence of tinnitus and hyperacusis in individuals with FHP with and without chronic neck pain compared with controls.

**Methods:**

Fifty individuals with FHP were included and classified into two groups according to the presence of nonspecific chronic neck pain: FHP with neck pain and FHP‐only. Fifty individuals without FHP or neck pain constituted the control group. Tinnitus presence was recorded in all participants. Hearing levels were assessed using pure‐tone audiometry, and subjective hearing difficulties were evaluated with the Turkish version of the Amsterdam Inventory for Auditory Disability and Handicap (T‐AIADH). Central auditory processing was examined using the Modulation Detection Test (MDT) and the Temporal Fine Structure–Adaptive Frequency (TFS–AF) test. Sound tolerance was assessed using the Khalfa Hyperacusis Questionnaire.

**Results:**

No significant differences were observed among the groups in pure‐tone hearing thresholds, MDT, TFS–AF, or hyperacusis scores (*p* > 0.05). However, T‐AIADH scores were significantly higher in the FHP with neck pain group than in the other groups (raw *p* = 0.022; Holm‐adjusted *p* = 0.044). Tinnitus prevalence was also significantly higher in this group (*p* = 0.003).

**Conclusion:**

This study did not detect significant differences in objective auditory measures among individuals with FHP‐only and controls. However, individuals with FHP and neck pain reported greater subjective hearing difficulties and a higher prevalence of tinnitus, highlighting the importance of considering neck pain in FHP evaluation.

## Graphical abstract

Individuals with forward head posture and chronic neck pain reported greater self‐perceived auditory difficulties and a higher prevalence of tinnitus. However, objective auditory measures, including pure‐tone thresholds, temporal fine structure, and modulation detection, did not significantly differ between groups.

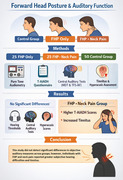

## 1 Introduction

Forward head posture (FHP) is defined as an excessive anterior positioning of the head relative to the shoulders, with the external auditory canal located anterior to the coronal plane (Arooj et al. 2022). FHP results in increased compressive forces on the cervical apophyseal joints (Khayatzadeh et al. 2017). Prolonged adaptation to this abnormal posture leads to elongation of the deep cervical flexor and infrahyoid muscles, while shortening occurs in the suprahyoid muscles and cervical extensors (Arooj et al. 2022). Consequently, FHP imposes increased mechanical stress on the muscles, tendons, and ligaments supporting the cervical spine, thereby predisposing individuals to various cervical pathologies, including spinal degeneration (Straker et al. 1997; Hong et al. 2019; Kirupa et al. 2020).

The development of this postural disorder may be influenced by several factors, including prolonged smartphone and computer use, sleeping with the head in an elevated position, and insufficient cervical muscle strength. Untreated FHP has been associated with trigger point formation in the suboccipital muscles, neural sensitization, headaches, and various musculoskeletal and neuromuscular disorders, such as temporomandibular joint dysfunction (Singla and Veqar 2017; Lee et al. 2014). Moreover, recent evidence has reported an association between FHP and neck pain, particularly in adult and older populations, indicating that FHP increases the risk of developing neck pain (Mahmoud et al. 2019).

While sound transmission and neural encoding are mediated by the peripheral auditory system, higher‐order processes such as sound interpretation, speech‐in‐noise perception, and sound localization/lateralization are carried out by the central auditory system (Litovsky 2015). Dysfunction within these systems or in anatomically and functionally related structures may lead not only to peripheral or central hearing disorders but also to conditions such as tinnitus and reduced sound tolerance (Henry et al. 2014). Such impairments can adversely affect communication abilities, social participation, and overall quality of life.

The functional mechanisms of the auditory system have been extensively investigated and are largely well defined. Recent neurophysiological evidence indicates that auditory processing does not rely solely on auditory inputs; rather, information from the somatosensory and visual systems modulates auditory signal processing along the ascending auditory pathway (Wu et al. 2015). This multisensory interaction begins at the cochlear nucleus, the earliest central relay of the auditory system. Proprioceptive and mechanosensory inputs originating from the neck, jaw, face, and scalp are conveyed to the cochlear nucleus primarily via the trigeminal ganglion and spinal trigeminal nucleus (Sp5), as well as through the dorsal root ganglia and dorsal column nuclei (Wu et al. 2015; Haenggeli et al. 2005; Zeng et al. 2011). While these somatic inputs predominantly terminate in the granule cell domain of the cochlear nucleus, a subset directly projects to the fusiform cell layer of the dorsal cochlear nucleus, thereby contributing to early‐stage auditory–somatosensory integration (Gómez‐Nieto and Rubio 2011).

In addition to central auditory–somatosensory interactions, another important pathway through which cervical and neck pathologies may influence the auditory system involves peripheral and vascular mechanisms (Karam et al. 2021). Cervical spine disorders can alter vertebral artery hemodynamics, leading to reduced vascular supply to the inner ear, or impair cochlear perfusion through mechanical compression of neural structures and the vertebral artery caused by osteophytic formations. Such vascular and neurovascular compromise may result in elevated hearing thresholds, tinnitus, or the coexistence of both symptoms (Karam et al. 2021; Vasaghi‐Gharamaleki and Naser 2017).

In the existing literature, the effects of neck problems and neck pain on auditory symptoms such as hearing loss and tinnitus have been addressed in several studies (Karam et al. 2021; Vasaghi‐Gharamaleki and Naser 2017; Koning and Meulen 2022). However, the role of FHP in auditory function has largely been overlooked. Only a limited number of studies have examined the effect of FHP on pure‐tone hearing thresholds (Fallahian et al. 2023). Nevertheless, auditory function is a multidimensional construct that extends beyond peripheral hearing thresholds and encompasses central auditory processing (CAP) abilities. Therefore, the aim of the present study was to describe and compare auditory function in individuals with FHP according to the presence or absence of accompanying chronic neck pain. Accordingly, pure‐tone hearing thresholds, CAP measures, self‐reported auditory difficulties, and the presence of tinnitus and hyperacusis were assessed.

## 2 Methods

### 2.1 Participants and Ethical Considerations

This study was designed as a cross‐sectional comparative study and was conducted among individuals who were presented to the neurosurgery outpatient clinic. The study was reported in accordance with the Strengthening the Reporting of Observational Studies in Epidemiology (STROBE) statement, and the completed STROBE checklist is provided as File S1. Ethical approval was obtained from the İzmir Bakırçay University Non‐Interventional Clinical Research Ethics Committee (decision no: 2651; study no: 2639). Written and verbal informed consent was obtained from all participants prior to inclusion.

All participants were evaluated by an experienced neurosurgeon, and detailed medical histories were obtained. During this evaluation, participants’ current complaints, history and duration of neck pain, previous ear or neck trauma, known otological, neurological, or psychiatric disorders, and use of ototoxic medications were questioned. All participants then underwent otoscopic examination (PulseMed, Türkiye), tympanometry (226 Hz probe tone; Neurosoft, Audio‐SMART, Russia), and pure‐tone audiometry. In individuals with neck pain, magnetic resonance imaging (MRI) findings were also reviewed as part of the clinical evaluation. The exclusion criteria are presented below.

Exclusion criteria for all participants:

- age below 18 years or above 50 years,
- history of otological, neurological, or psychiatric disorders,
- history of ear or neck trauma,
- otalgia,
- use of ototoxic medications,
- tympanic membrane perforation detected on otoscopic examination,
- Type B or Type C tympanogram on tympanometric evaluation,
- hearing loss detected on pure‐tone audiometry.

Additional exclusion criteria for individuals with neck pain:

- neck pain not meeting the chronicity criterion, defined as pain lasting at least 3 months,
- MRI findings indicating disc herniation, radiculopathy, myelopathy, or other specific cervical pathologies requiring surgical intervention.

**FIGURE 1:**
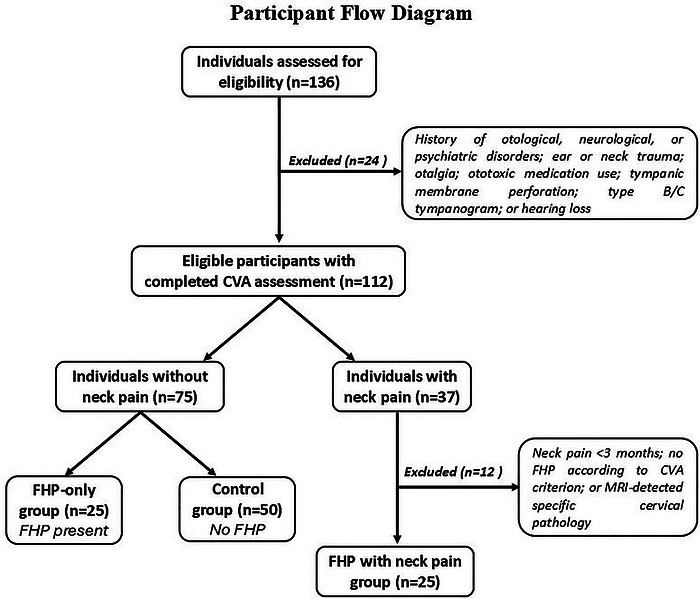
Participant flow diagram of the study. A total of 136 individuals were assessed for eligibility. Twenty‐four individuals were excluded according to the general exclusion criteria. After the exclusion process, craniovertebral angle (CVA) assessment was completed in 112 eligible participants. Of these, 75 individuals had no neck pain and 37 individuals had neck pain. Among individuals without neck pain, 25 participants who met the CVA criterion for forward head posture (FHP) were included in the FHP‐only group, whereas 50 individuals who did not meet the CVA criterion for FHP were included in the control group. Among individuals with neck pain, 12 were excluded. The remaining 25 participants were included in the FHP with neck pain group.

Following these evaluations, the craniovertebral angle (CVA) was measured. Accordingly, 25 individuals with FHP who had nonspecific chronic neck pain (> 3 months) were assigned to the FHP with neck pain (FHP & neck pain) group. Twenty‐five individuals who did not report neck pain or a history of chronic or recurrent neck pain but met the CVA criterion for FHP constituted the FHP‐only group. In addition, 50 individuals without FHP or neck pain were included as the control group. The participant flow diagram of the study is presented in Figure 1. Individuals with neck pain were recruited from patients presenting to the neurosurgery outpatient clinic with a complaint of neck pain, whereas individuals in the FHP‐only and control groups were recruited from the patients’ relatives. Demographic characteristics, including age, sex, comorbidities, height, and weight, were recorded for all participants.

### 2.2 Procedure

#### 2.2.1 Assessment of FHP

**FIGURE 2:**
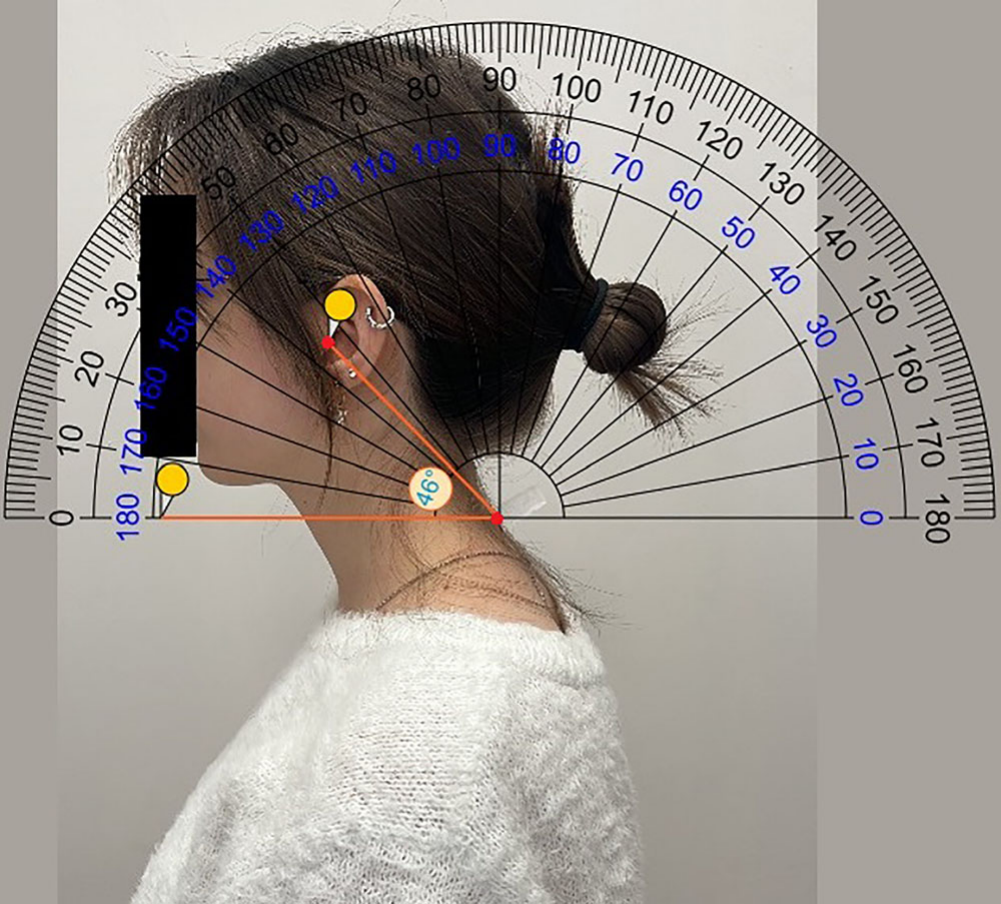
Measurement of the craniovertebral angle (CVA) using an online goniometer.

FHP was assessed using the CVA method based on photographic analysis. Anatomical landmarks, including the tragus of the ear and the spinous process of the seventh cervical vertebra (C7), were identified and marked. Participants were photographed from the left sagittal plane while standing upright and maintaining their natural posture, using a digital camera positioned at shoulder height. The CVA was calculated as the angle formed between a horizontal reference line passing through C7 and a line extending from C7 to the tragus. A CVA value of < 45.5° was considered indicative of FHP (Mostafaee et al. 2024). To ensure measurement reliability and reproducibility, angle measurements were performed using an online goniometer software (available at: https://www.ginifab.com/feeds/angle_measurement/) (Figure 2).

#### 2.2.2 Neck Disability Index (NDI)

The NDI is a 10‐item questionnaire that was adapted into Turkish by Kesiktaş et al. (2012), with its validity and reliability previously established (Kesiktas et al. 2012). Each item is scored on a scale ranging from 0 to 5, with higher total index scores indicating greater levels of disability. The internal consistency of the total index score was assessed using Cronbach's alpha coefficient and reported as 0.88.

#### 2.2.3 Determination of Hearing Thresholds

Pure‐tone audiometry was performed using a MAICO MA 25 screening audiometer (Maico Diagnostics, Germany). Air‐conduction thresholds were obtained using TDH‐39 supra‐aural headphones across the frequency range of 250–8000 Hz. The pure‐tone average (PTA) was calculated as the mean of thresholds at 500, 1000, 2000, and 4000 Hz. A PTA value greater than 25 dB HL was considered indicative of hearing loss.

### 2.3 Assessment of Tinnitus

Participants who reported tinnitus completed the Turkish version of the Tinnitus Handicap Inventory (THI) (Aksoy et al. 2007). The THI is a valid and reliable instrument used to assess the degree of handicap associated with tinnitus. The internal consistency and reliability of the Turkish version have been reported with a Cronbach's alpha coefficient of 0.88 and an intraclass correlation coefficient (ICC) ranging from 0.78 to 0.90 (Aksoy et al. 2007). The inventory consists of 25 items, with responses scored as “Yes” (four points), “Sometimes” (two points), or “No” (zero points). Higher total scores indicate a greater negative impact of tinnitus on the individual's quality of life.

### 2.4 Psychometric Assessment of Auditory Performance

Psychometric auditory performance was evaluated using the Turkish version of the Amsterdam Inventory for Auditory Disability and Handicap (T‐AIADH), which was developed to assess individuals’ subjective evaluations of various listening situations encountered in daily life (Mujdeci et al. 2016). The inventory demonstrates excellent internal consistency, with a reported Cronbach's alpha coefficient of 0.98, and consists of 30 items grouped into five subdomains: distinction/identification of sounds, localization of sounds, speech intelligibility in quiet, speech intelligibility in noise, and detection of sounds. Participants rated how frequently they experienced each auditory situation using a four‐point response scale: “almost never” (0), “occasionally” (1), “frequently” (2), and “almost always” (3). The total score ranges from 0 to 90, with higher scores indicating greater perceived auditory difficulty or disability.

### 2.5 Assessment of Hyperacusis

Reduced sound tolerance was assessed using the Turkish version of the Khalfa Hyperacusis Questionnaire (KHQ) (Erinç and Derinsu 2020). The questionnaire demonstrates good internal consistency, with a reported Cronbach's alpha coefficient of 0.81. It consists of two sections, of which only the second section is scored. The scored section includes 14 items, each rated on a 5‐point scale ranging from 0 to 4, yielding a maximum total score of 42. Higher total scores indicate greater severity of reduced sound tolerance.

### 2.6 Assessment of CAP

CAP abilities were assessed using the Modulation Detection Test (MDT) and the Temporal Fine Structure–Adaptive Frequency (TFS–AF) test, administered via the Psychoacoustics software on a Windows 10–based computer and delivered through Sennheiser HDA 200 headphones (Sęk and Moore 2020). The number of reversal points was set to *n* = 8, and threshold values were calculated in accordance with the software documentation as the mean of the stimulus values at the final (*n* − 4) reversal points. All tests were conducted using a two‐alternative forced‐choice (2AFC) paradigm with an adaptive two‐down one‐up procedure.

For the MDT, participants were required to identify the interval containing amplitude modulation. A 1‐kHz carrier tone was presented with a fixed modulation frequency of 9 Hz, while only the modulation depth (*m*) was adaptively varied. Thresholds were calculated in decibels (20·log_10_(*m*)), with lower threshold values indicating better modulation sensitivity.

The TFS–AF test assessed binaural temporal fine structure (TFS) sensitivity based on the perception of interaural phase differences (IPDs). The IPD was fixed at 180°, and the carrier frequency was adaptively increased starting from 200 Hz. The threshold was defined as the highest frequency at which the participant was able to detect the IPD change, with higher threshold frequencies reflecting better binaural TFS sensitivity.

### 2.7 Sample Size Calculation and Statistical Analysis

An a priori sample size calculation was performed using G*Power version 3.1.9.4 for a one‐way ANOVA with three independent groups. Based on a pilot study, an effect size of *f* = 0.382 was used. With an alpha level of 0.05 and 80% power (*β* = 0.20), the minimum required sample size was calculated as 72 participants in total, corresponding to 24 participants per group.

Statistical analyses were performed using IBM SPSS Statistics for Windows, version 21.0 (IBM Corp., Armonk, New York, USA). The normality of data distribution was assessed using the Shapiro–Wilk test. Data with a normal distribution are presented as mean ± standard deviation, whereas non‐normally distributed data are reported as median (minimum–maximum) [Q1–Q3]. Group comparisons were conducted using the Kruskal–Wallis test, and when significant differences were detected, pairwise post hoc comparisons were performed using Dunn's test with Bonferroni correction. To reduce the risk of Type I error due to multiple outcome comparisons, Holm–Bonferroni correction was applied within predefined outcome families. Specifically, the audiological test family included pure‐tone hearing thresholds, MDT, and TFS–AF scores. The main questionnaire outcome family included KHQ and the T‐AIADH total score. Questionnaire subdomain analyses were considered exploratory and corrected separately within the subdomain family. Categorical variables were analyzed using the chi‐square test or Fisher's exact test, depending on expected cell frequencies. Associations between variables were examined using Spearman's rank correlation analysis. A *p* value of < 0.05 was considered statistically significant for all analyses.

## 3 Results

There were no significant differences among the groups in terms of age, sex, weight, height, body mass index (BMI), smoking status, alcohol consumption, or comorbidities (*p* > 0.05). The demographic and clinical characteristics of the groups are presented in Table 1.

**TABLE 1:** Demographic and clinical characteristics of the study groups.

Variable	FHP & neck pain group	FHP‐only group	Control group	*p*	Post‐hoc *p*
**Age (years)**	23.24 ± 7.72 (18–46)	24.12 ± 7.93 (17–42)	24.20 ± 7.93 (17–45)	0.972	
**Gender**				0.246	
Male, *n* (%)	8 (32.0)	7 (28.0)	23 (46.0)		
Female, *n* (%)	17 (68.0)	18 (72.0)	27 (54.0)		
**CVA**	43 (34–45)	40.00 (32–45)	50 (46–56)	< 0.001	0.591^a^ < 0.001^b^ < 0.001^c^
**Height (cm)**	170 (155–189) [163–178]	170 (155–187) [165–178]	165.5 (150–187) [160–178]	0.538	
**Weight (kg)**	69 (50–91) [58–76]	65 (42–90) [57–80]	59 (41–100) [54–76]	0.282	
**BMI (kg/m^2^)**	23.53 (18.31–30.07) [21.14–25.61]	21.89 (16.87–33.46) [19.23–25.47]	21.74 (17.10–33.03) [19.78–24.62]	0.418	
**Smoking, *n* (%)**	8 (32.0)	9 (36.0)	16 (32.0)	0.934^d^	
**Alcohol use, *n* (%)**	3 (12.0)	2 (8.0)	4 (8.0)	0.833^e^	
**Comorbidities**					
Diabetes mellitus, *n* (%)	1 (4.0)	2 (8.0)	1 (2.0)		
Hypertension, *n* (%)	0 (0.0)	0 (0.0)	1 (2.0)		
Hyperlipidemia, *n* (%)	1 (4.0)	1 (4.0)	1 (2.0)		
Cardiovascular disease, *n* (%)	1 (4.0)	2 (8.0)	2 (4.0)		

^a^FHP & neck pain group versus FHP group.

^b^FHP group versus control group.

^c^FHP & neck pain group versus control group.

^d^Chi square.

^e^Fisher exact.

No significant differences were observed among the groups regarding air‐conduction pure‐tone hearing thresholds or PTA (*p* > 0.05). Group‐specific air‐conduction thresholds and PTA values are shown in Table 2.

**TABLE 2:** Pure‐tone hearing thresholds and pure‐tone air‐conduction average according to groups.

Variable	FHP & neck pain group median (min–max) [Q1–Q3]	FHP‐only group median (min–max) [Q1–Q3]	Control group median (min–max) [Q1–Q3]	*p*	Effect size, *η* ^2^ *H* (95% CI)
**250 Hz (dB HL)**	15 (0–35) [10–20]	15 (0–30) [10–20]	10 (0–40) [10–15]	0.386	0.000 (0.000–0.109)
**500 Hz (dB HL)**	20 (5–35) [15–25]	15 (0–40) [10–20]	15 (0–35) [10–20]	0.131	0.021 (0.000–0.147)
**1000 Hz (dB HL)**	15 (0–30) [10–20]	15 (0–30) [10–20]	15 (0–40) [10–20]	0.875	0.000 (0.000–0.063)
**2000 Hz (dB HL)**	10 (0–20) [5–10]	5 (0–15) [5–10]	10 (0–25) [5–10]	0.459	0.000 (0.000–0.094)
**4000 Hz (dB HL)**	10 (0–25) [5–10]	5 (0–15) [5–10]	5 (0–40) [5–10]	0.354	0.001 (0.000–0.094)
**6000 Hz (dB HL)**	10 (0–25) [5–15]	10 (0–20) [5–15]	10 (0–40) [5–10]	0.260	0.007 (0.000–0.124)
**8000 Hz (dB HL)**	5 (0–30) [0–10]	5 (0–35) [0–10]	5 (0–35) [3.75–10]	0.349	0.001 (0.000–0.103)
**PTA**	12.50 (1.25–22.50) [8.75–17.50]	10.00 (2.50–21.25) [8.75–15.00]	11.25 (0–25) [7.50–15.31]	0.413	0.000 (0.000–0.098)

Tinnitus was present in six individuals (24%) in the FHP & neck pain group, one individual (4%) in the FHP‐only group, and one individual (2%) in the control group. The prevalence of tinnitus was significantly higher in the FHP & neck pain group compared with the other groups (*p* = 0.003). THI scores were available only for participants who reported tinnitus and were therefore presented descriptively. Among participants with tinnitus, the THI score was 23.42 ± 15.77 in the FHP & neck pain group (*n* = 6), 12 in the FHP‐only group (*n* = 1), and 8 in the control group (*n* = 1).

No significant difference was found among the groups in terms of KHQ scores (*p* = 0.079) (Table 3). However, a significant difference was observed in T‐AIADH scores among the groups (raw *p* = 0.022; Holm‐adjusted *p* = 0.044) (Table 3). Post hoc analyses revealed that the T‐AIADH scores of the FHP & neck pain group were significantly higher than those of the other two groups (adjusted *p* < 0.05). The speech intelligibility in noise subdomain showed an unadjusted group difference (raw *p* = 0.017), with higher scores in the FHP & neck pain group. However, this difference did not remain statistically significant after Holm–Bonferroni correction within the subdomain family (adjusted *p* = 0.085).

**TABLE 3:** THI and KHQ scores across groups.

Variable	FHP & neck pain group median (min–max) [Q1–Q3]	FHP‐only group median (min–max) [Q1–Q3]	Control group median (min–max) [Q1–Q3]	*p* (Holm‐adjusted *p*)	Post‐hoc *p*	Effect size, *η* ^2^ *H* (95% CI)
**KHQ**	10 (1–33) [8–15]	10 (0–35) [6–13]	8 (0–26) [4–12]	0.079		0.032 (0.000–0.161)
**T‐AIADH**	23 (0–45) [7–28]	6 (0–39) [2–18]	8.5 (0–81) [3–18]	0.022 (0.044)	0.046^a^ 1.000^b^ 0.038^c^	0.053 (0.000–0.196)
Distinction of sounds	4 (0–13) [1–5]	0 (0–7) [0–3]	1 (0–21) [0–3]	0.070		0.034 (0.000–0.169)
Localization of sounds	5 (0–10) [1–7]	1 (0–11) [0–4]	1 (0–15) [0–4]	0.065		0.036 (0.000–0.179)
Speech intelligibility in quiet	4 (0–10) [1–6]	1 (0–8) [0–4]	2 (0–15) [0–4]	0.061		0.037 (0.000–0.167)
Speech intelligibility in noise	5 (0–11) [4–8]	2 (0–12) [0–6]	3 (0–15) [2–6]	0.017 (0.085)	0.034^a^ 1.000^b^ 0.032^c^	0.052 (0.000–0.195)
Detection of sounds	2 (0–8) [1–5]	1 (0–7) [0–2]	1 (0–15) [0–2]	0.174		0.015 (0.000–0.138)

^a^FHP & neck pain group versus FHP group.

^b^FHP group versus control group.

^c^FHP & neck pain group versus control group.

In the FHP & neck pain group, the median duration of neck pain was 16.24 months (range: 3–120), and the median NDI score was 11.24 (range: 4–24). A moderate positive correlation was found between NDI scores and T‐AIADH scores in this group (*r* = 0.50, *p* = 0.010).

No significant differences were observed among the groups in terms of TFS–AF or MDT scores (*p* > 0.05). Group‐specific TFS–AF and MDT results are presented in Table 4.

**TABLE 4:** TFS–AF and MDT thresholds across groups.

Variable	FHP & neck pain group median (min–max) [Q1–Q3]	FHP‐only group median (min–max) [Q1–Q3]	Control group median (min–max) [Q1–Q3]	*p*	Effect size, *η* ^2^ *H* (95% CI)
**TFS–AF (Hz)**	940.1 (212.1–2860.4) [323.0–1350.1]	654.0 (218.4–2860.4) [312.9–1142.5]	940.0 (212.1–2860.4) [388.8–1522.8]	0.302	0.004 (0.000–0.113)
**MDT (dB)**	−18.93 (−25.71 to −3.55) [−22.16 to −9.56]	−18.60 (−24.74 to −5.17) [−20.14 to −12.31]	−18.28 (−28.94 to −4.20) [−22.56 to −10.48]	0.927	0.000 (0.000–0.058)

## 4 Discussion

The aim of the present study was to describe and compare auditory functions in individuals with FHP according to the presence or absence of accompanying nonspecific chronic neck pain. To this end, individuals with FHP were divided into two groups based on the presence or absence of nonspecific chronic neck pain, and their pure‐tone hearing thresholds, CAP measures, self‐reported auditory difficulties, tinnitus, and hyperacusis were compared with those of controls. The findings showed that the FHP & neck pain group reported higher self‐reported auditory difficulties than the FHP‐only and control groups, although the effect size was small. Tinnitus was also more prevalent in this group. In contrast, no meaningful group differences were observed in pure‐tone hearing thresholds, hyperacusis, or CAP test outcomes.

FHP is one of the most common postural deviations in the general population and is estimated to occur at a clinically relevant magnitude in approximately two‐thirds of individuals (Vakili et al. 2016; Oakley et al. 2024). The CVA, which is widely used in the assessment of FHP, is also considered a reliable indicator of functional neck disability (Aimi et al. 2019). Despite claims suggesting an association between FHP and neck pain, the nature of this relationship remains controversial (Mahmoud et al. 2019; Silva et al. 2010). Some studies have reported no significant differences in FHP between individuals with and without neck pain (Silva et al. 2010). In contrast, a recent meta‐analysis identified age as an important confounding factor in the relationship between FHP and neck pain, demonstrating a significant association particularly in older adults (Mahmoud et al. 2019). In the present study, no significant difference in CVA was observed between individuals in the FHP & neck pain group and those in the FHP‐only group. This finding may be related to age effects, as suggested by the meta‐analysis, given that the study sample predominantly consisted of young adults.

FHP alters cervical biomechanics by increasing extension and lordosis at the C0–C2 segments, while leading to increased flexion and reduced lordosis at the C2–C7 segments (Demirtaş and Güzel 2025). However, unlike other cervical conditions (Karam et al. 2021; Vasaghi‐Gharamaleki and Naser 2017), the relationship between FHP and auditory function has not been adequately explored. Fallahian et al. (2023) classified individuals with FHP into mild, moderate, and severe groups and compared their pure‐tone air‐conduction hearing thresholds with those of a control group. The authors reported that FHP did not affect pure‐tone air‐conduction thresholds. In the present study, in addition to pure‐tone hearing thresholds, CAP measures, self‐reported auditory difficulties, tinnitus, and hyperacusis were examined in individuals with FHP. No meaningful differences were observed in objective auditory measures between the FHP‐only group and controls. Therefore, within the limitations of this cross‐sectional study, FHP alone did not appear to be associated with detectable differences in auditory function.

Nevertheless, several studies have examined the relationship between FHP and auditory function in the context of hearing loss (de Souza Melo et al. 2013). de Souza Melo et al. (2013) reported a higher prevalence of FHP in adolescents with sensorineural hearing loss compared with their normal‐hearing peers. The authors suggested that dizziness symptoms may play a mediating role in the association between hearing loss and FHP. Accordingly, the increased frequency of dizziness in individuals with hearing loss may lead to compensatory behaviors aimed at stabilizing the head in a specific position, thereby promoting the development of FHP. However, the technology use habits of individuals with hearing loss, such as smartphone use, may differ from those of their healthy peers (Aktas Kucuktas et al. 2021). Thus, the higher prevalence of FHP in this population may also be attributable to excessive use of such devices. Therefore, to better elucidate the relationship between auditory function and FHP, further longitudinal studies with bidirectional designs are warranted.

Cervical pathologies often cause pain, leading to movement avoidance and restricted mobility. Several studies have investigated the relationship between neck pain and auditory function (Karam et al. 2021; Vasaghi‐Gharamaleki and Naser 2017). Vasaghi‐Gharamaleki and Naser (2017) examined pure‐tone air‐conduction hearing thresholds in individuals with neck pain and reported elevated hearing thresholds—particularly at speech frequencies—especially in males and in those with left‐rotation limitation compared with controls. Similarly, Karam et al. (2021) reported that restricted left cervical rotation in individuals with cervical spondylosis was an independent predictor of hearing loss, particularly in males. These findings suggest that dysfunctions arising in the upper cervical region may affect the vascular and neural structures supplying the ear, thereby contributing to hearing loss (Vasaghi‐Gharamaleki and Naser 2017). In contrast, no significant differences in pure‐tone hearing thresholds were observed among the groups in the present study. However, individuals in the FHP & neck pain group exhibited poorer subjective auditory performance compared with the other two groups. Notably, no differences were found between the FHP‐only group and the control group in either pure‐tone hearing thresholds or subjective hearing abilities.

The T‐AIADH, which was used to evaluate participants’ subjective auditory abilities, assesses the five hearing functions defined by the World Health Organization (WHO): distinction of sounds, localization of sounds, speech intelligibility in quiet, speech intelligibility in noise, and detection of sounds (Mujdeci et al. 2016). These functions are predominantly mediated by the central auditory system. In this context, it is noteworthy that although individuals in the FHP & neck pain group exhibited poorer T‐AIADH scores, no significant differences were observed among the groups in behavioral CAP test outcomes. The MDT evaluates an individual's ability to process temporal envelope information, which is critical for speech intelligibility and speech perception in noise (Jain and Sahoo 2014). The TFS–AF test, on the other hand, reflects spatial hearing and binaural integration abilities by assessing the use of TFS information based on interaural time differences (Moore and Sek 2009). Therefore, the poorer subjective auditory performance observed in the FHP & neck pain group may be perceptual rather than indicative of a structural or functional auditory deficit. Chronic pain has been shown to induce increased somatic awareness, attentional bias toward pain, and central sensitization (De Meulemeester et al. 2023; Eccleston et al. 1997). Such processes may lead individuals to perceive their auditory performance as poorer than it objectively is, thereby influencing subjective hearing assessments independently of objective auditory measures.

Tinnitus is defined as the perception of sound in the absence of an external acoustic stimulus. This perception can be distressing and may lead to reduced quality of life, limitations in daily activities, difficulties with concentration, sleep disturbances, and an increased psychological burden. Tinnitus originating from the neck and cervical region is referred to as somatic tinnitus and has been examined in greater detail compared with other neck‐related auditory disorders (Koning and Meulen 2022; Aydoğan et al. 2022; Ralli et al. 2017). In a retrospective study evaluating 61 patients with tinnitus, Koning and Meulen (2022) reported that activation of proprioceptive input pathways associated with cervical pain may contribute to the development of tinnitus as well as hearing loss in the 250 Hz–4 kHz frequency range. The authors proposed that increased proprioceptive input secondary to cervical pain may influence the cochlear nucleus—particularly via the cuneate nucleus—thereby leading to low‐ to mid‐frequency hearing loss and increased tinnitus severity. The observation that pain management modalities such as transcutaneous electrical nerve stimulation (TENS), when applied to the cervical region, can alleviate tinnitus symptoms (Aydoğan et al. 2022) supports the hypothesis proposed by Koning and Meulen (2022). Consistent with these findings, the present study demonstrated a higher prevalence of tinnitus in individuals in the FHP & neck pain group compared with both the FHP‐only and control groups. This finding suggests that the development of tinnitus may be more strongly associated with chronic neck pain than with FHP itself.

Hyperacusis is a reduced sound tolerance disorder characterized by the perception of everyday environmental sounds, which are normally tolerated by healthy individuals, as excessively loud, uncomfortable, or unbearable (Anari et al. 1999). In the present study, KHQ scores were higher in the FHP & neck pain group compared with the other groups; however, this difference did not reach statistical significance. The relatively higher KHQ scores observed in the FHP & neck pain group may be partly explained by the higher prevalence of tinnitus in this group, as hyperacusis and tinnitus frequently co‐occur. It has been reported that approximately 40% of patients with tinnitus also experience hyperacusis (Anari et al. 1999). In addition, increased central sensitization associated with chronic neck pain (De Meulemeester et al. 2023) may have contributed to altered sound tolerance levels in these individuals.

The present study is among the first to adopt a multidimensional approach to examining auditory functions in individuals with FHP. Our findings suggest that self‐reported auditory difficulties and tinnitus may be more prominent when FHP is accompanied by neck pain, rather than with FHP alone. In this regard, our study may be considered a preliminary investigation providing exploratory evidence on the relationship between FHP, neck pain, and auditory function. Future studies including larger sample sizes and older adult populations are warranted to more comprehensively examine the long‐term associations between FHP and auditory functions.

This study has several limitations. First, participants with neck pain were recruited from individuals presenting to a neurosurgery outpatient clinic. Therefore, this group represents a care‐seeking and symptom‐aware population rather than the general population. This recruitment source may have increased the likelihood of symptom reporting and may limit the generalizability of the findings to community‐based individuals with FHP. Second, MRI was reviewed only for participants with neck pain as part of their clinical evaluation. Routine MRI was not performed in the FHP‐only and control groups. Although these participants were screened through detailed medical history and clinical examination, the presence of undetected asymptomatic cervical pathology cannot be completely ruled out. This may have introduced potential bias in group comparisons, particularly regarding the exclusion of specific cervical pathologies.

## 5 Conclusion

Our findings suggest that FHP alone was not associated with meaningful differences in objective auditory measures within the scope of the present study; however, individuals with FHP accompanied by neck pain reported greater self‐reported auditory difficulties and showed a higher prevalence of tinnitus. The absence of meaningful group differences in pure‐tone hearing thresholds, CAP tests, and hyperacusis may suggest that auditory complaints in these individuals are more closely related to self‐reported perceptual and symptom‐based factors rather than to detectable differences in objective auditory measures. These findings highlight the importance of considering neck pain in the evaluation of individuals with FHP.

## Author Contributions

**Kaan Tuğberk Özdemir**: methodology, project administration, resources, supervision, data curation, writing – review and editing, validation, investigation, conceptualization, funding acquisition . **Emre Söylemez**: conceptualization, investigation, funding acquisition, writing – original draft, visualization, validation, methodology, formal analysis, software, data curation, resources.

## Funding

The authors have nothing to report.

## Disclosure

Patients were not actively involved in the design or conduct of this study. They were informed about the study content at the beginning and were provided with information regarding the study results at the end.

## Ethics Statement

This research was conducted according to the principles of the Declaration of Helsinki and was approved by the Ethics Committee of the Izmir Bakırcay University (decision no: 2651/10.12.2025; study no: 2639). Participants gave informed consent to participate in the study before taking part.

## Conflicts of Interest

The authors declare no conflicts of interest.

## Supporting information



Supplementary Materials: brb371611‐sup‐0001‐SuppMat.doc

## Data Availability

The data that support the findings of this study are available from the corresponding author upon reasonable request.
